# Refractory Amiodarone-Induced Thyrotoxicosis Requiring the Consideration of Thyroidectomy: A Case Report and Review of Management Challenges

**DOI:** 10.7759/cureus.114586

**Published:** 2026-08-16

**Authors:** Hishaam A Yunas, Lakshey Mahajan, Khine Wut Yee Lwin, Shehr B Syeda

**Affiliations:** 1 Diabetes and Endocrinology, Wye Valley NHS Trust, Hereford, GBR

**Keywords:** amiodarone, carbimazole, diabetic ketoacidosis, thyroidectomy, thyrotoxicosis

## Abstract

Amiodarone-induced thyrotoxicosis (AIT) is an uncommon but clinically significant complication of amiodarone therapy that may be associated with substantial morbidity, particularly in patients with underlying cardiovascular disease. We report the case of a 61-year-old gentleman with a history of atrial fibrillation, ischaemic heart disease, heart failure, and obesity, who developed severe thyrotoxicosis after approximately 3.5 years of amiodarone treatment. Routine thyroid function monitoring showed stable thyroid levels for three years; however, despite an abnormality identified at a later stage, the patient remained asymptomatic. Shortly after, he was admitted with vomiting, abdominal discomfort, poor mobility, weight loss, and a free thyroxine (fT4) level exceeding 100 pmol/L. Carbimazole therapy was initiated, but persistent thyrotoxicosis necessitated dose escalation, discontinuation of amiodarone, and commencement of prednisolone for suspected AIT. During a subsequent admission, the patient developed euglycaemic diabetic ketoacidosis and later steroid-induced diabetes, requiring treatment and both diabetic and endocrine team involvement. Thyroid ultrasound demonstrated normal-low vascularity without focal abnormalities, and thyroid autoantibodies were negative. Although thyroid function tests initially improved with combined medical therapy, biochemical response later plateaued with a fT4 level of 52 pmol/L at the end of month 8, prompting referral for thyroidectomy. This case describes a challenging presentation of AIT with an incomplete response to conventional medical management and progression to consideration of definitive surgical treatment.

## Introduction

Amiodarone is a class III antiarrhythmic agent widely used in the management of atrial and ventricular arrhythmias. Due to its high iodine content and direct effects on thyroid physiology, amiodarone is associated with a range of thyroid abnormalities, including hypothyroidism and amiodarone-induced thyrotoxicosis (AIT) [1].

AIT is an uncommon but potentially serious complication that can be challenging to diagnose and manage, particularly in patients with underlying cardiovascular disease, whereby uncontrolled thyrotoxicosis may exacerbate cardiac dysfunction and increase morbidity. Two principal forms of AIT have been described: type 1, resulting from iodine-induced excess thyroid hormone synthesis, and type 2, a destructive thyroiditis leading to the release of preformed thyroid hormones [2]. Distinguishing between these subtypes is fundamental, as treatment strategies differ and mixed forms may occur. There is no definitive test to distinguish between the two types; this remains a significant clinical challenge.

We present a case of AIT in a patient with atrial fibrillation and significant cardiovascular comorbidity who demonstrated a suboptimal response to conventional medical therapy and was subsequently considered for definitive surgical management.

## Case presentation

We present a 61-year-old gentleman with a background of ischaemic heart disease, heart failure, and obesity, who was eventually diagnosed with AIT following a readmission with abnormal thyroid function tests (TFTs).

Having been diagnosed with atrial fibrillation in early 2022, the patient was initially started on amiodarone following direct-current cardioversion. Whilst on amiodarone, his TFTs were regularly monitored, which remained stable until late 2025 whereby a shift in his thyroid-stimulating hormone (TSH) level was noted on his blood results (Table 1). The patient was asymptomatic at the time. Not long after, at the end of 2025, he was admitted with vomiting, abdominal discomfort, poor mobility, and weight loss. Bloods taken in the emergency department demonstrated thyrotoxicosis, with a free thyroxine (fT4) of more than 100 pmol/L. He was subsequently referred to the endocrinology team and was commenced on a once-daily dose of carbimazole 30 mg. Although severe thyrotoxicosis can exacerbate cardiovascular issues, the decision to continue amiodarone was made in view of its long half-life.

**Table 1:** Relevant blood results during the diagnostic process from the onset of abnormal thyroid markers until the most recent endocrinology outpatient appointment TSH: thyroid-stimulating hormone; fT4: free thyroxine; fT3: free triiodothyronine; TRAb: thyroid-stimulating hormone receptor antibodies; TPOAb: thyroid peroxidase antibodies; HbA1c: glycated haemoglobin; pH: potential of hydrogen

Serum tests	Day 1	Month 3	Month 5	Month 7	Month 8 (early)	Month 8 (middle)	Month 8 (end)	Reference range
TSH (mU/L)	2.12	0.33	<0.01	<0.01	<0.01	<0.01	<0.01	0.27-4.20
fT4 (pmol/L)	N/A	N/A	90.5	>100	57.3	43.0	52	12-22
fT3 (pmol/L)	N/A	N/A	17.8	7.1	6.6	N/A	6.3	3.9-6.7
TRAb (IU/L)	N/A	N/A	N/A	N/A	1.5	N/A	N/A	0-2.1
TPOAb (IU/mL)	N/A	N/A	8	N/A	7	N/A	N/A	0-35
HbA1c (mmol/mol)	40	42	N/A	N/A	N/A	71	73	26-41
pH (pH units)	N/A	N/A	7.27	7.35	7.36	N/A	N/A	7.35-7.45
Glucose (mmol/L)	N/A	N/A	11.0	9.1	8.4	N/A	N/A	3.6-5.3
Ketones (mmol/L)	N/A	N/A	2.3	1.0	N/A	N/A	N/A	0-0.6

In early 2026, he was readmitted with hypotension and euglycaemic diabetic ketoacidosis (DKA), having presented with symptoms of abdominal pain and vomiting, with raised ketones and metabolic acidosis noted on a venous blood gas (Table 1). He was subsequently started on a variable rate insulin infusion, and his dapagliflozin was held. This led to an improvement in the patient's symptoms and biochemistry. Since there was no improvement in the TFTs from his previous admission, carbimazole was uptitrated to 60 mg once daily. The decision to discontinue amiodarone was eventually made due to the severity of deterioration in thyroid function and readmission. In light of a suspected diagnosis of AIT, the patient was started on prednisolone 30 mg once daily. Whilst on prednisolone, the patient developed steroid-induced diabetes, which warranted a review by the diabetic team. Upon review, he was started on metformin and gliclazide. His blood glucose levels were closely monitored as well.

**Figure 1:**
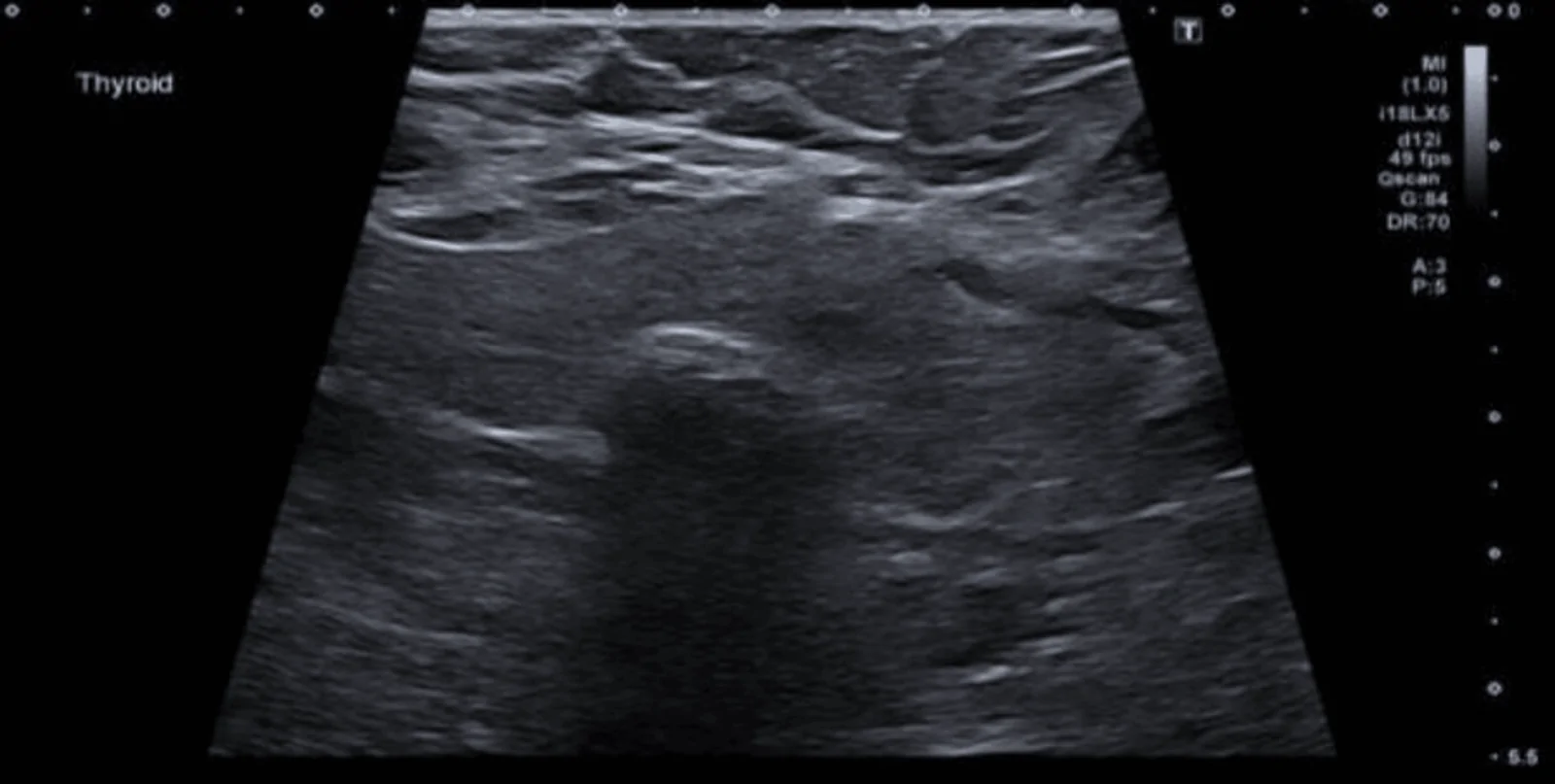
Thyroid ultrasound scan, reported as unremarkable

Over the course of six weeks, there was an initial improvement in his TFTs, which later plateaued. His thyroid ultrasound scan with Doppler flow demonstrated a normal, smooth texture, with no focal lesion or change in echogenicity and a normal-low level vascularity (Figure 1). His thyroid antibodies were negative. In view of the suboptimal response to medical management, plans for a thyroidectomy were discussed with the patient in an ear, nose, and throat (ENT) clinic. The patient consented to be added to the waiting list for the thyroidectomy. At the time of writing this report, the patient remains on the waiting list. In the interim, he has continued taking carbimazole 40 mg once daily. His prednisolone was tapered off, and both endocrinology and diabetes clinic follow-ups have been arranged.

## Discussion

AIT is an important and potentially life-threatening complication of long-term amiodarone therapy [1]. Although amiodarone is highly effective in the management of atrial fibrillation and other tachyarrhythmias, its high iodine content and direct cytotoxic effects on thyroid follicular cells predispose patients to significant thyroid dysfunction [2]. Thyroid abnormalities may occur months to years following the initiation of therapy and, importantly, can persist despite cessation of the drug due to its prolonged half-life and accumulation within adipose tissue [3].

This case highlights several clinically significant aspects of AIT, including delayed presentation, diagnostic uncertainty with subtype classification, resistance to conventional medical therapy, and associated metabolic complications. Our patient developed severe biochemical thyrotoxicosis approximately three years after the commencement of amiodarone despite previously stable thyroid function monitoring. This emphasises the necessity for continued long-term surveillance of thyroid function in patients receiving chronic amiodarone therapy, even in the absence of symptoms [4].

AIT is classically categorised into two distinct subtypes. Type 1 AIT results from iodine-induced excess thyroid hormone synthesis, typically occurring in individuals with pre-existing thyroid pathology such as multinodular goitre or latent Graves' disease. In contrast, type 2 AIT represents a destructive thyroiditis characterised by the release of preformed thyroid hormones secondary to direct follicular injury. Distinguishing between the two entities is clinically important, as management differs considerably. However, differentiation can be challenging in practice, and mixed forms are increasingly recognised (Table 2) [1,5,6].

**Table 2:** Differences between AIT type 1 and AIT type 2 AIT: amiodarone-induced thyrotoxicosis; USG: ultrasound sonography; RAIU: radioiodine uptake; TSI: thyroid-stimulating immunoglobulin; TRAb: thyroid-stimulating hormone receptor antibody

	Type 1	Type 2
Mechanism	Excessive thyroid hormone production due to iodine overload	Destructive thyroiditis leading to the release of preformed thyroid hormones into circulation
Pre-existing thyroid disease	Patients may have latent autoimmune thyroid disease	Pre-existing thyroid disease absent
Prevalence	Most common type in iodine-deficient regions	Most common type in iodine-sufficient regions
Imaging	Normal/increased vascularity on USG. RAIU scan shows variable uptake	Decreased vascularity on USG. RAIU scan shows low/absent uptake
Labs	May have positive TSI and TRAb. Normal/increased thyroglobulin	No detectable thyroid antibodies. Low thyroglobulin
Treatment	Antithyroid medications	Steroids

In this patient, several features favoured a diagnosis of type 2 AIT with a mixed AIT uncertainty. Thyroid ultrasound with Doppler demonstrated normal thyroid architecture with low-normal vascularity and no nodularity, whilst thyroid autoantibodies were negative. Furthermore, the delayed onset following prolonged amiodarone exposure and partial biochemical response to glucocorticoid therapy supports a destructive inflammatory process rather than autonomous hormone overproduction. Nevertheless, due to persistent thyrotoxicosis and the overlap frequently observed between AIT subtypes, combination therapy with high-dose carbimazole and prednisolone was initiated [5].

Management of AIT presents a significant therapeutic challenge, particularly in patients with underlying cardiovascular disease. Uncontrolled thyrotoxicosis may precipitate arrhythmias, thus worsening heart failure and haemodynamic instability. Although cessation of amiodarone is often considered, the decision must be made on a case-by-case basis given its prolonged half-life and the potential risk of recurrent cardiac arrhythmia [1,5]. In this case, amiodarone was initially continued due to the patient's cardiac background before eventually being discontinued following deterioration in thyroid function and readmission [7].

An additional complexity in this presentation was the development of euglycaemic DKA. The patient was prescribed dapagliflozin for heart failure with reduced ejection fraction, which likely contributed to the metabolic derangement. Sodium-glucose cotransporter-2 (SGLT2) inhibitors are increasingly recognised as a cause of euglycaemic DKA, particularly during periods of acute physiological stress [8]. Severe thyrotoxicosis itself promotes insulin resistance, enhanced lipolysis, and increased hepatic gluconeogenesis, thereby contributing to ketogenesis [9]. It is therefore likely that the coexistence of uncontrolled thyrotoxicosis and SGLT2 inhibitor therapy acted synergistically to precipitate the patient's presentation. Furthermore, glucocorticoid therapy subsequently contributed to steroid-induced hyperglycaemia, necessitating diabetic team involvement and the initiation of appropriate therapy [10,11].

Despite escalation of medical therapy, the patient's thyroid function demonstrated only partial improvement with subsequent biochemical plateauing. This illustrates the refractory nature that can occur in severe AIT. The frequency of medical therapy failure in AIT is well documented. Isaacs et al. reported that initial medical therapy achieved euthyroidism in only 52% of a cohort of 66 patients, with the poorest outcomes observed in those requiring combination treatment, likely reflecting greater biochemical severity at presentation [12,13]. Thyroidectomy was ultimately required in 33% of that cohort, consistent with the Mayo Clinic experience in which medically refractory disease was the most common surgical indication [14]. The 2018 European Thyroid Association guidelines formally recommend thyroidectomy without delay in patients with AIT unresponsive to medical therapy or with deteriorating cardiac function [1]. This case therefore sits within a well-recognised pattern of refractory AIT, and the referral for thyroidectomy represents guideline-concordant management.

## Conclusions

This case highlights the diagnostic and therapeutic challenges associated with AIT, particularly in patients with significant cardiovascular comorbidities. Despite regular thyroid function monitoring and prompt initiation of antithyroid therapy, the patient exhibited persistent biochemical thyrotoxicosis requiring escalation of treatment, corticosteroid therapy, and eventual referral for thyroidectomy. The patient still remains on the waiting list so there are no post-operative outcomes available. The development of steroid-induced diabetes further underscores the complexity of managing AIT and the potential adverse effects of treatment. Clinicians should maintain a high index of suspicion for AIT in patients receiving amiodarone, even when initially asymptomatic, and adopt a multidisciplinary approach involving endocrinologists, cardiologists, diabetes specialists, and surgeons when medical therapy fails to achieve adequate disease control. Early recognition and individualised management are essential to optimise outcomes and reduce the risk of complications.
